# Use of In-Situ ESR Measurements for Mechanistic Studies of Free Radical Non-Catalytic Thermal Reactions of Various Unconventional Oil Resources and Biomass

**DOI:** 10.3390/ijms252011047

**Published:** 2024-10-15

**Authors:** Hajra Maqsood, Basim Abu-Jdayil, Joy H. Tannous

**Affiliations:** Chemical & Petroleum Engineering Department, F1 Building, United Arab Emirates University, Al-Ain P.O. Box 15551, United Arab Emirates

**Keywords:** free radicals, electron spin resonance, heavy oil, coal, biomass, thermal reactions, oxidation reactions

## Abstract

The exhaustion of conventional light oils necessitates the shift towards unconventional sources such as biomass, heavy oil, oil shale, and coal. Non-catalytic thermal cracking by a free radical mechanism is at the heart of the upgrading, prior to refining into valuable products. However, thermal pyrolysis is hindered by the formation of asphaltenes, precursors to coke, limiting cracking, causing equipment fouling, and reducing product stability. Free radicals are inherently present in heavy fractions and are generated during thermal processes. This makes these reactive intermediates central to understanding these mechanisms and limiting coking. Electron spin resonance (ESR) spectroscopy facilitates such mechanistic studies. Over the past decade, there has been no review of using in-situ ESR for studying thermal processes. This work begins with a brief description of free radicals’ chain reactions during thermal reactions and the wealth of information ESR provides. We then critically review the literature that uses ESR for mechanistic studies in thermal pyrolysis of biomass, heavy oil, shales, and coal. We conclude that limited literature exist, and more investigations are necessary. The key findings from existing literature are summarized to know the current state of knowledge. We also explicitly highlight the research gaps.

## 1. Introduction

Due to the exhaustion of conventional light oil reserves during the 1970s oil crisis, there has been a significant focus on unconventional oil sources such as oil sands bitumen, heavy oil, coal, and shale oil in the global energy sector [1,2]. In order to meet the growing demand for lighter fuels, and prior to refining into specific petroleum products, it is required to upgrade these unconventional sources. In the case of heavy oil upgrading, non-catalytic thermal cracking, that consists of the fractional conversions of heavier crude oils, lies at the heart of the process [3]. In fact, free radical non-catalytic thermal cracking of heavy fractions is crucial in order to meet product specifications and be able to transport it through pipelines. In addition to the challenge of upgrading, these oil fractions suffer from instability due to their free radical oxidation upon exposure to oxygen or during storage. Both non-catalytic cracking and oxidation take place through a free radical mechanism. The pyrolysis of coals, oil shales, and biomass is also dominated by a free radical mechanism that determines the success of the cracking process.

Free radicals are the molecular species which contain an unpaired electron within an atomic orbital and have the tendency to exist independently and donate or accept an electron [4]. Free radicals are highly unstable and reactive species inherently present in petroleum and generated during thermal conversion reactions of petroleum and biofuels [5]. Free radicals can be broadly classified into two categories: volatile or reactive radicals and stable radicals [6]. Volatile free radicals are unstable, move freely, and react in nanoseconds, whereas stable ones can be studied without specific precautions, such as molecular oxygen (O_2_) [7]. In addition, free radicals inherently present in petroleum, oil shales, biomass, and coal and that have stability of geological scales, are called persistent. The dynamic formation and consumption of free radicals during non-catalytic reactions may have a destructive impact on the feedstocks and their associated processes such as extraction, upgrading, and refining [8].

One of the major drawbacks of unconventional sources upgrading processes dominated by a free radical mechanism is that some free radicals are susceptible to undergo addition reactions contributing to the formation of high molecular weight substances like asphaltenes, organic wax, and emulsions which in turn, form aggregates and coke [9,10,11]. During the thermal cracking of petroleum and biofuels, certain covalent bonds undergo thermal cleavage, including those between C-O, C-S, C_al_-C_al_, and C_ar_-C_al_ [12]. In addition to the inherently present free radicals, thermal bond cleavage generates additional free radicals that undergo free radical reactions, which can be sequential and/or parallel, during the thermal upgrading process. In other words, radicals produced by the breakdown of large and small oil molecules tend to interact and propagate either independently or in conjunction with other radicals. Low molecular weight products formed by cracking reactions are desirable. However, free radicals also undergo addition reactions that lead to the formation of coke and asphaltenes, which restrict the cracking process, clogs the units and reduces the stability of the product [13,14]. Moreover, oxidation reactions may also occur due to the presence of dissolved oxygen in crude oil during the thermal cracking procedure. It initiates the auto-catalytic free radical chain reactions that generate peroxyl radicals which further react to form hydroperoxides. Hydroperoxides form more reactive radical species such as alkoxyl and hydroxyl. Thus, these radical species further contribute to the aromatization and condensation of large radical fragments into coke and reduces the oil cracking process. Therefore, it is crucial to understand the free radical mechanism, reaction pattern and the progression of free radicals throughout the coke induction phase to control the formation of coke and improve the thermal upgrading processes of unconventional resources.

As the importance of measuring and controlling free radicals in these processes is clear as described above, many advancements have been made in analytical techniques paired with powerful software for the analysis and characterization of these intermediate species. For example, Fourier-Transform Ion Cyclotron Resonance Mass Spectrometry (FTICR-MS), Nuclear Magnetic Resonance (NMR), and Infrared Spectroscopy (IR) are utilized, but their applications are restricted to the structural analysis of compounds [10]. However, the utilization of electron spin resonance (ESR) spectroscopy has facilitated the investigation of free radical precursors [15]. It is a spectroscopic technique utilised for the direct detection of the electron Zeeman transition that is expressed in substances having at least one unpaired electron, specifically, the paramagnetic species. These species consist of organic free radicals, ions of transition metals, triplet-state molecules, solid point-defects such as the F-centre, systems containing conducting electrons like metals and semiconductors, and charge-transfer complexes [16]. These species exhibit paramagnetism and are, thus, ESR active. They are chemically reactive and participate in numerous chemical reactions [17]. The major advantage of ESR over other analytical techniques is that it is not limited to the structural examination, but it also has the ability to detect the spin states of paramagnetic species, the chemical neighborhood of the free radical, and the number of spins which offers invaluable information. This can assist in following the free radical reactions with time, temperature, and other parameters. Thus, ESR can be effectively applied to the detection and quantification and, thus, control of free radicals during processes dominated by a free radical mechanism including thermal cracking and oxidation reactions of various unconventional oil sources [18].

**Figure 1:**
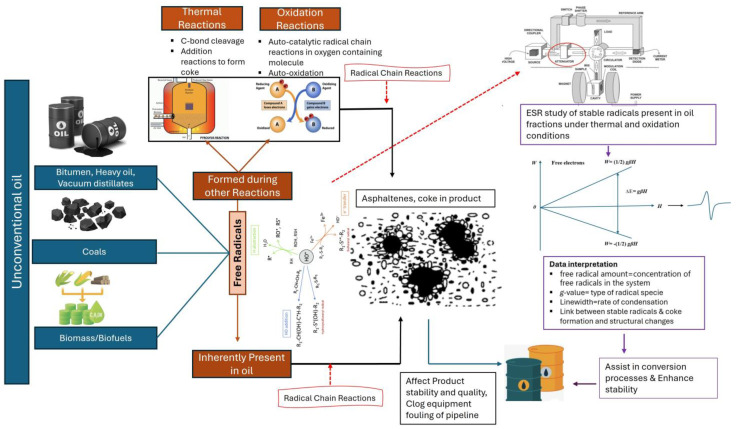
Summary of the article scope.

Understanding thermal cracking reactions is of great importance and is, to date, not established. Though ESR has been used for in-situ investigation of free radical mechanisms in thermal cracking reactions of oil, literature on the use of ESR for in-situ measuring and tracking of free radicals in oil systems have not been reviewed to identify the gaps and suggests future work in the field. In the literature related to coal, Zhou et al. provided a concise overview of coal conversion procedures utilizing ESR up till 2019 [17]. The aim of the current review article is to provide an exhaustive summary of literature, in the last decade, that used ESR for in-situ investigation of free radical reactions in non-catalytic thermal cracking of petroleum, biomass, oil shale, and coal. To provide context, we start with a brief explanation of the chain radical reactions during thermal cracking highlighting the importance of understanding these mechanisms. We then explain how ESR could be used in-situ to (i) detect and quantify free radicals, (ii) find coupling and condensation reaction rates that generate coke by calculating the reaction kinetics, and free radicals quenching through hydrogen donors such as DHP [19,20,21], and (iii) to provide insights into the free radical pathways associated with thermal reactions [22]. In the current work, we also identify the research gaps necessary to understand the thermal cracking of the various investigated feedstocks, which assist in optimizing these processes and prevent the formation of undesirable products. The summary of this review article is illustrated in (Figure 1) below. In Figure 1, we illustrate that unconventional oil resources such as bitumen, heavy oil, vacuum distillate, coal, and biomass consist of significant amounts of free radicals that are either present inherently or formed through other reactions (e.g., thermal or oxidation reactions) during cracking processes. These reactions involved in the formation of heavy compounds like asphaltenes, and coke affect the product stability, quality, and clog refinery’s equipment and cause fouling of pipelines. To cope with this problem, ESR spectroscopy can be used as an advanced technique for the in-situ study of free radicals, their concentration, type, rate of condensation, their link with coke formation, and its structural change in oil systems which can further assist in the conversion processes and product stability. Thus, the summary shows the significance of understanding free radicals and mechanisms through the in-situ study assisted by ESR in unconventional oils.

## 2. Role of Chain Radical Reactions in Thermal Cracking

Thermal cracking involves the breakdown and modification of hydrocarbon molecules under elevated temperatures. It is the prevailing technique utilized to decrease viscosity. The thermal cracking of heavier fractions results in the formation of light valuable products and other by-products during the thermal upgrading. These residual by-products reduce product yield and quality, and demand more oil input comparable to the amount of product [23].

Thermal cracking process involves a free radical mechanism typically divided into initiation, propagation, and termination during the pyrolysis of crude oil [24]. The initiation step involves the cleavage of certain covalent bonds (e.g., α-C_al_ and β-C_al_ in C_ar_-C_al_-C_al_ chains and C_al_-O, C-C, C-S, C_al_-C_al_, C_al_-C_ar_), resulting in the formation of two free radicals and triggering free-radical reactions [25]. The formed radicals tend to propagate and condense either independently or with other radicals through sequential and parallel free radical reactions, which cause either chain growth or chain transfer indicating the propagation and termination steps, respectively. During the chain growth, these free radicals produce large radical fragments. The frequency with which these reactions repeat itself is known as the chain length; whereas the termination reactions include chain transfer of free radicals through coupling which forms neutral compounds. In the gas-phase reaction, coupling happens as a heavy by-product or neutral compound may be required to assist in the dissipation of the heat generated by the exothermic reaction [23]. This results in the development of asphaltene and coke, by free radical addition reactions [26]. Thermal cracking also initiates oxidation reactions, generating alkyl and peroxyl radicals due to the dissolved atmospheric oxygen during the process of the radical chain reaction. These reactions are auto-catalyzed during oil conversion which can lead to severe oxidation reactions due to higher oxygen consumption by oil and turn the actual feed into oxygenated oil. Numerous organic by-products of autoxidation, including alcohols, aldehydes, ketones, and esters, accumulate until the reaction ceases; termination reactions of radicals occur [27]. Certain molecules within the medium are destructive and have the ability to combine to produce products with a large molecular mass such as coke, which fundamentally alter physical properties of crude oil [22].

Thus, the non-catalytic thermal cracking process and additional auto-catalyzed oxidation reaction during the cracking of oil, coal, and other resources results in the development of certain heavier substances that separate and deposit as solid particles, which is considered an undesirable product [28,29]. Products with high propensity to separate into solid particles are known as asphaltenes or aggregates. Asphaltenes are high molecular weight substances distinguished by the existence of condensed poly-aromatic rings resulting from the the combination of several mono-aromatic rings. They are classified as a solubility class (i.e., dissolve in light aromatics like toluene but insoluble in *n*-alkane). They are commonly recognized for containing a low H:C atomic ratio. Coke formation resulting from phase separation of asphaltenes during thermal cracking is characterized by Wiehe’s phase separation model [30]. It was proposed that during thermal conversion, additional free radical reactions produce asphaltenes, which, in turn, due to their high molecular weight, become insoluble in the bulk oil and form coke [31]. Coking results in the formation of deposits that can cause obstructions in equipment and have a detrimental impact on subsequent processes. An increased concentration of these compounds elevates the probability of fouling of pipeline, product instability, and cracking operation. This is a significant disadvantage of thermal processes because they produce by-products that are heavier than the feed which restricts the degree of cracking, such as thermal severity, and reduces the stability of the products [13,14]. Clogging and product instability has also economic consequences.

**Figure 2:**
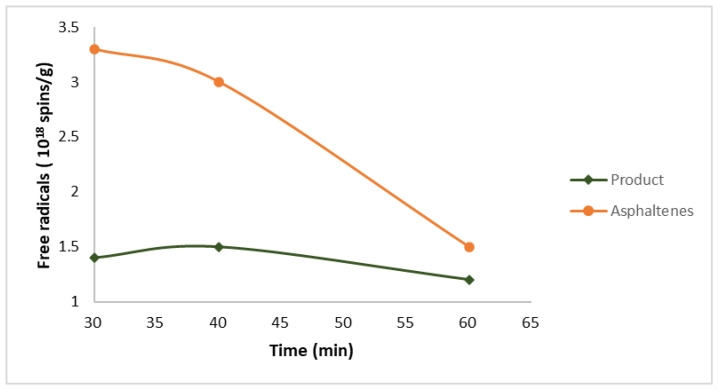
Free radical concentration in deasphalted oil and in the thermally converted product of the deasphalted oil at 400 °C, namely asphaltenes and total product [35].

In order to prevent formation of undesirable heavy molecular weight components in these processes, it is important to understand the free radical mechanism [32]. Free radicals can be present inherently in dense fractions, such as bitumen and vacuum residues, even prior to any thermal conversion process, or they could be generated due to heat [33]. Thus, the process of initiation is not compulsory for the generation of free radicals in thermal conversions. It was confirmed through the feed characterization of deasphalted oils that they contain 0.9 × 10^18^ spins/g of free radicals [34]. To ascertain whether pyrolysis temperature induces a modification in the free radical content, the same oil was subjected to thermal transformation at 400 °C, and the free radical content of the generated residues (i.e., products and asphaltenes) was determined. Results indicated that the concentrations of free radicals are significantly higher in the products (1.4 × 10^18^) compared to the input material (0.9 × 10^18^). The second observation was that the asphaltenes contained a greater quantity of free radicals (3.3 × 10^18^) than the total product which contained 1.4 × 10^18^ spins/g of free radicals after thermal conversion, thus proving that free radical reactions are highly associated with heavy compound formation [35]. As shown in (Figure 2), the asphaltenes contain highly radical concentration as compared to the feed and the generated product. Siddiquee et al. suggested that the asphaltenes contain high aromatic substances that are major sources of free radical reactions [36].

Though the relationship between free radical content variation and asphaltenes formation is not fully established yet, it will be further discussed in Section 4 below. Literature is saturated with studies linking the process’s operating conditions to the amount of asphaltenes or coke formed. During non-catalytic thermal cracking, a higher temperature is required for the pyrolysis of feed because of the higher activation energy required for the cracking. Asphaltenes and coke generated from pyrolysis reactions largely depend on the feedstock selection, operational parameters, and the system selected for pyrolysis.

**Figure 3:**
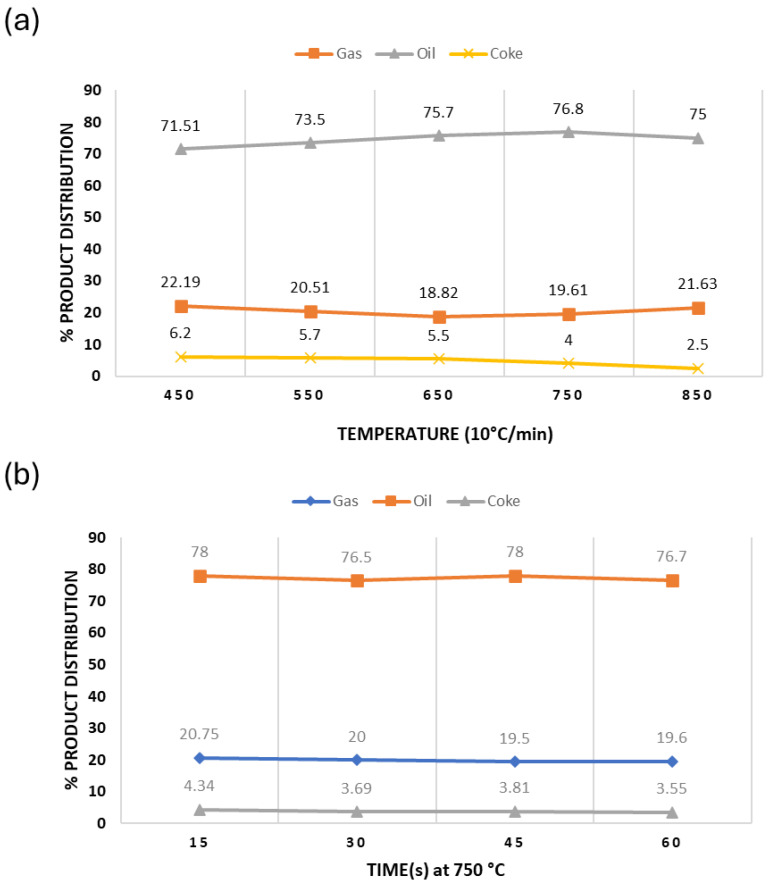
Percentage of gas, oil, and coke yield in the thermally converted product of raw oil sludge under (**a**) different temperatures (**b**) different reaction times. This graph shows the result generated by Jin et al. [38]. In (**a**) the *x*-axis denotes the temperature ranging from 450 to 850 °C which is increasing at the rate of 10 °C/min. and the values of data points denoting the % product distribution.

The extent of asphaltenes formation is related to the process severity of the thermal cracking reaction, namely temperature and time. For instance, an investigation was conducted in 2020, [37] to examine the thermal decomposition of Arabian light (AL) crude oils at three distinct temperatures: 600, 625, and 650 °C. The conversion rate was significantly impacted by the reaction temperature which represents the direct correlation between conversion and temperature. Another study using oil sludge as feed illustrated the relationship between reaction temperature and time with respect to product distribution as shown in (Figure 3) [38]. Four observations can be made here: (i) at constant temperature, heat duration regulates the conversion and the production of selective products, (ii) at a pyrolysis temperature of 750 °C, the product yield varies as time changes, (iii) though oil sludge already contains a large amount of oxidized material, it still produces coke during thermal cracking, and (iv) the coke weight percentage decreases as the temperature and time increases but is still above the threshold allowed to avoid fouling or clogging. In fact, as temperature increased, the coke formation decreased from 6.30 wt% to 2.68 wt% due to complex gas-solid reactions which lead to the initiation of secondary cracking of volatiles [39,40].

**Figure 4:**
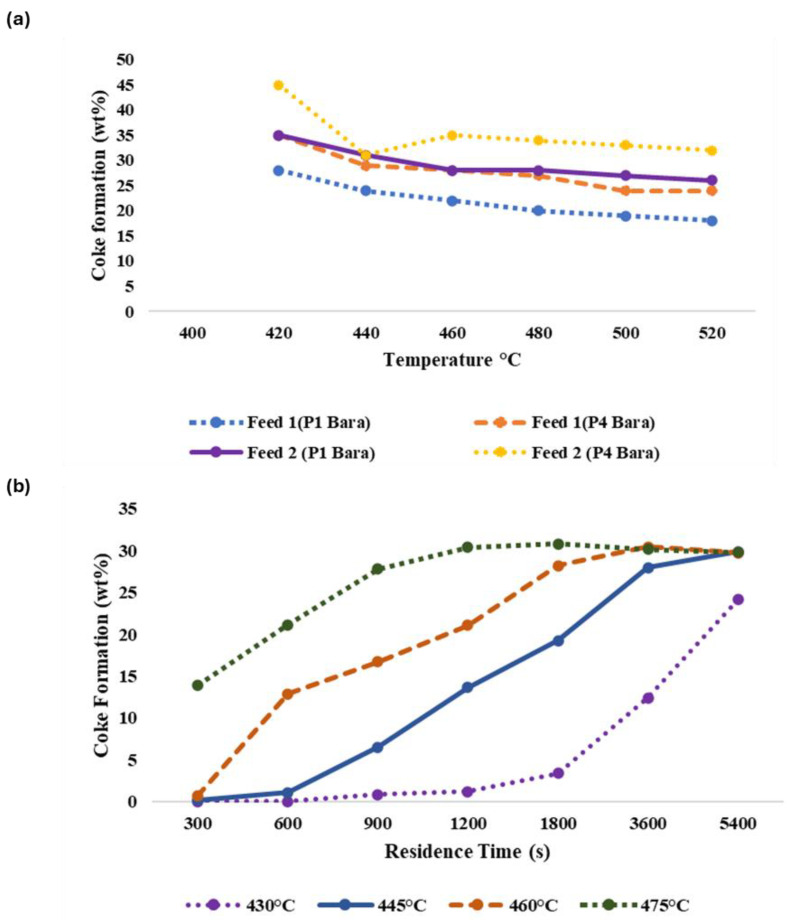
Wt% of coke in thermally converted products of 2 vacuum residues (**a**) at different temperature ranging at pressures (1 and 4 Bara (Bar absolute)) [41], (**b**) at different residence time for various temperatures [42].

Other studies also showed that coke yield decreases as the temperature increases [41] but the opposite behavior is observed when the feed is subjected to variable residence times [42]. It shows that an increase in residence time increases coke formation (Figure 4).

The previously cited literature, along with other works on oilsands bitumen thermal cracking [43,44], shows intolerable wt% of asphaltenes and coke found in the collected products that are in the acceptable range.

It is postulated that the variations in the weight percentage of asphaltenes and coke is due to the free radical reactions. It was observed that some of the radicals that escaped the coupling process start their propagation and termination step and cause an increase in asphaltene content. On the other hand, when asphaltenes content is reduced, it shows that some of the free radicals combine, condense, polymerize, and convert into the coke thus reducing the asphaltene contents. Both processes (i.e., formation of asphaltenes and coke) occur simultaneously. Thus, asphaltenes and coke wt%, per se, cannot explain the free radical mechanism occurring. It is, therefore, necessary to gain insights into free radical mechanisms by other tools such as using electron spin resonance (ESR) spectroscopy for in-situ measurements.

## 3. Electron Spin Resonance (ESR) as a Powerful Tool for Studying Free Radical Mechanisms

**Figure 5:**
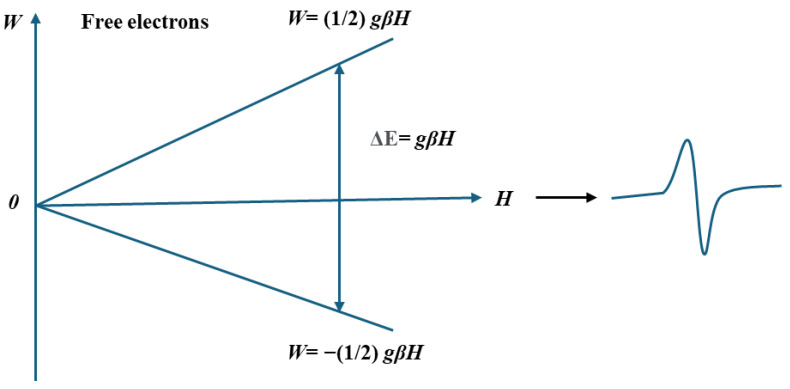
Spin movement of unpaired electrons upon absorption of magnetic field.

Electron spin resonance (ESR) spectroscopy is an analytical method with high-resolution capabilities used to examine unpaired electrons, known as paramagnetic species, including organic free radicals, conducting electrons, F-center, triple state molecule, and transition metal ions in the system [16]. ESR spectroscopy quantifies the energy absorption by unpaired electrons as they transition between different spin states in the presence of a magnetic field. It is an effective method utilized in the detection of free radicals and the environment in which they exist. A single line is generated by the free radical, which corresponds to the transition spin state ±1/2. The energies of the two potential configurations (i.e., spin up +1/2 and spin down −1/2) of an unpaired electron in the presence of an external magnetic field are distinct [45], as illustrated by the energy level diagrams in (Figure 5). When no external magnetic field is applied, the energy of the two spin states is equivalent [46].

The interaction between a free electron and neighboring nuclei with non-integer spin numbers, like ^1^H, leads to hyperfine splitting of the ESR spectrum. This splitting is similar to the impact of spin-spin coupling on an NMR spectrum. The electron spin resonance (ESR) spectra of crude oils emits signals from two distinct entities, specifically, the vanadyl group VO^2+^ and free radicals, both can be identified from ESR spectra [47,48]. ESR spectrum of vanadium (IV) and carbon-centered radicals do not overlap, therefore it is possible to quantify both vanadium and organic radicals [33].

The relevance of ESR to the objective of this review is that it detects and quantifies free radicals inherently present in hydrocarbons and biomass or that can be generated upon bond breakage during oxidation reactions, pyrolysis, and other free radical processes [49]. It is worthwhile noting that *g*-value and line width are properties of great importance during the analysis of free radicals and are both obtained from ESR spectra.

### 3.1. g-Value

The constant Landé *g*-value for a compound defines the peak position and exhibits sensitivity to the chemical vicinity of the unpaired electron [49]. Most literature agree that coal and its products, with a higher *g*-value, have more heteroatomic radicals due to their stronger spin-orbit coupling interactions [50], while a lower *g*-value has more aromatic hydrocarbon radicals [51,52]. When heteroatoms are present in a non-localized system, the *g*-value tends to elevate due to the unpaired electrons’ movement over various atoms [47]. The absence of hyperfine splitting in oil sands bitumen generated samples [33], as well as other heavy materials like coal, has been attributed to factors such as signal overlap due to the presence of same radical species [18]. Therefore, in heavy fractions, the details of the arrangement of the free radicals which may be derived from hyperfine splitting are not preserved.

Free radical characteristics can be obtained through the average *g*-value [53]. There are various ranges of g-values for various centered free radicals. However, these ranges overlap, for instance, a *g*-value ranging from 2.0030 to 2.0040 signifies a compound comprising radicals centered on oxygen and carbon. Petrakis and Grandy, in 1980s, classified the free radical species present in different compounds of hydrocarbons depending on the *g*-value [54,55,56]. This classification can assist in differentiating free radical species in asphaltenes or coke that comprise the heavy fractions and possess the characteristics of a free radical mixture [57,58]. Pyrolysis of biomass and coal generate coke with free-radical having *g*-values ranging from 2.0027 to 2.0034 [52,53]. Coke that had *g*-values ranging from 2.0027 to 2.0029 was identified as containing carbon-centered radicals, whereas coke with *g*-values between 2.0026 and 2.0028 belonged to polycyclic aromatic radicals with low carbon-centered radicals. This shows again an overlap in the *g*-value ranges. If *g*-value exceeds 2.0040, the radicals are classified as oxygen-centered radicals [59].

**Figure 6:**
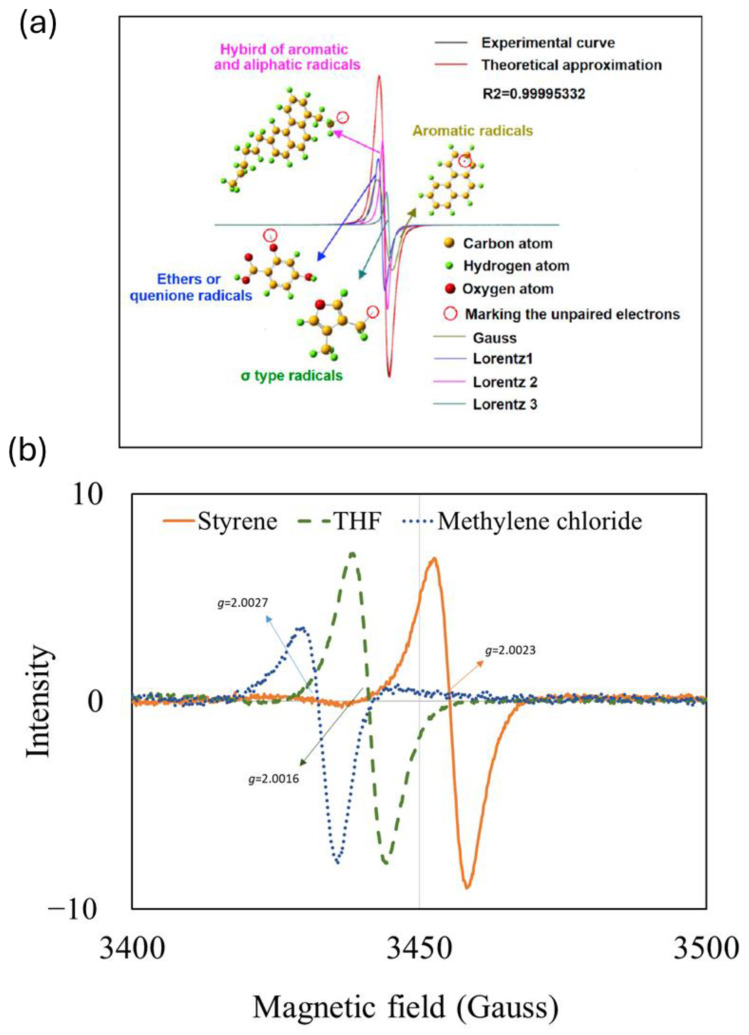
ESR spectra presenting different radical species which denotes different *g*-value (**a**). in coals studied by Liu et al. [60] and reprinted in [17] (**b**). Bitumen [64].

The importance of *g*-value is illustrated in Figure 6 below. ESR studies for the detection of stable radicals in superfine pulverized coals was conducted [60], and Figure 6a shows the ESR spectra generated by different radical species which indicate different *g*-value. On the other hand, (Figure 6b) shows the shift in the *g*-value when using various solvents for bitumen [61].

**Figure 7:**
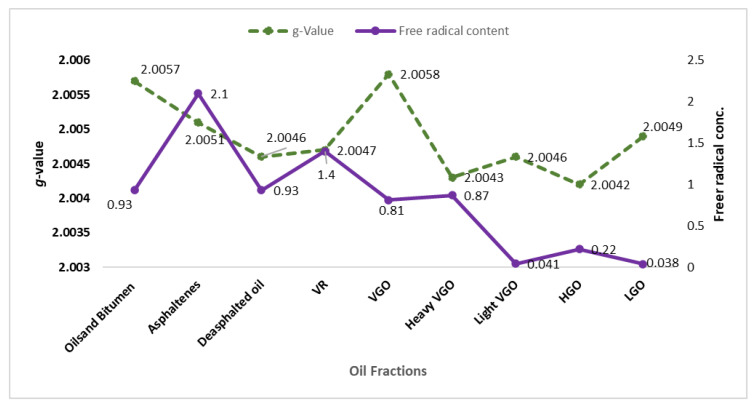
*g*-Value and free radical concentration in different oil fractions done by [65]. Purple line indicates the free radical concentration (×10^18^ spins/g), whereas, green line shows different *g*-values which are related to the type of radical species dominating in that specific fraction.

Multiple uses of (ESR) in the field of petroleum have been discovered [62]. Several researchers analyzed and measured organic free radicals in various petroleum fractions [63,64]. (Figure 7) shows the free radical concentration of different heavy fractions with their respective *g*-values [65].

**Figure 8:**
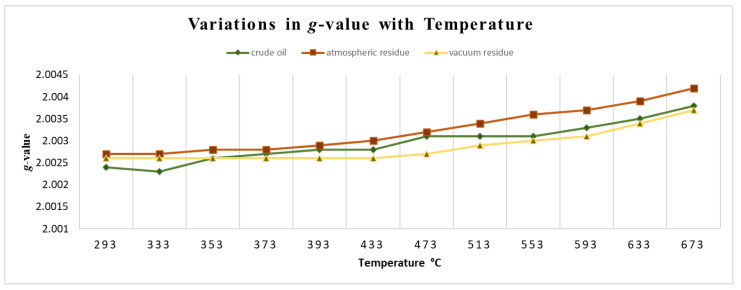
The change of *g*-value of various samples including atmospheric residue, vacuum residue, and crude oil during pyrolysis at different temperatures ranging from 292 to 673 K during the in-situ measurement using ESR [3].

Like other complex matrices, variations in the *g*-value in petroleum provide insights into the characteristics of the free radicals [47,66]. For example, the *g*-value was shown to be associated with the sulfur content in petroleum asphaltenes, along with the nitrogen, and oxygen heteroatom content [67]. An extensive correlation between ESR spectral data and reservoir parameters and oil properties can be demonstrated using the *g*-values [68,69]. Moreover, *g*-value is very sensitive to the chemical environment of the unpaired electron. Research was conducted [3] on different petroleum fractions including atmospheric residue, vacuum residue and crude oil which were subjected to thermal cracking under temperatures ranging from 293 to 673K. Results showed that the *g*-factors of all petroleum fractions undergoing thermal treatment exhibited a proportional relationship with temperature. It demonstrates that the heating method has the potential to cause alterations in the surrounding environment of free radicals when exposed to elevated temperatures which alternate the *g*-value as shown in (Figure 8).

### 3.2. Line Width

The spin-spin interaction between electron-proton and electron-electron, exchange effect, spin-lattice interaction, exchange and heteroatom effect, affect the linewidth of the ESR spectra [70]. The non-zero spin nuclei like hydrogen also have an effect by broadening the linewidth because zero-spin nuclei can absorb and emit the electromagnetic radiations which is detected by ESR and shows resonance which broadens the linewidth. This was proved by (i) increasing linewidth with hydrogen content of the hydrocarbon; (ii) decreasing linewidth with decreasing hydrogen content of chars during pyrolysis and oxidation [71,72], (iii) some compounds char having a larger linewidth than their deuterated precursors [71]. A higher concentration of unpaired electrons also creates a wider linewidth due to the higher interaction between electrons [52,55,73].

Spin-lattice interaction also broadens the line width by the transfer of energy to the surrounding lattice due to electrons’ exciting and de-exciting stages. This impact of spin-lattice interaction on line width is studied by Pilawa et al. [74] who observed increased linewidth due to higher spin-lattice interaction. It was also observed a narrowed linewidth due to opposite behavior of spin-lattice interaction [60], and minimal influence on line width because the spin-lattice interaction has a significantly longer relaxation period [75]. Duber et al. found relaxation times of 10^−9^–10^−7^ s for spin-spin interaction and 10^−6^–10^−4^ s for spin-lattice interaction in coals [42]. However, bridge bonds between aromatic clusters transmitting unpaired electrons, cause the exchange effect—which is the reason for linewidth narrowing—to lower result in broadening [70,76]. Some investigations suggested that heteroatoms expand the linewidth [30,43,77] and that anisotropy has a slight effect. The electron-proton/electron-electron/spin-lattice interactions and exchange effects are the main reasons for linewidth broadening or narrowing.

The presence of free radicals in crude oils is indicated by an unresolved line of approximately five Gauss in width in the ESR spectra [78]. The variation in linewidth between free radical peaks in different studies is contingent with the chemical composition of asphaltenes and paraffins [79]. Moreover, studies conducted on the relationship between temperature change and the line width of free radicals in heavy and extra-heavy crude oils can assist in the detection of changes in molecular mobility. When crude oil is subjected to elevated temperatures, its molecular mobility changes from vibrational to rotational motion. Due to the high molecular weight of heavy oils, they show less molecular mobility which causes the variation in linewidth difficult to observe; whereas lighter crude oils exhibit a broader line width of the ESR signal of organic free radicals due to high molecular mobility. Thus, heavier asphaltenes exhibit reduced mobility because their free electrons are interlocked. Therefore, linewidth alteration, if present, serves as an effective parameter for identifying alterations in the structure of heavy compounds; the line width enables us to investigate the impact on the molecular mobility of crude oil.

It is clear that ESR spectroscopy can be employed to analyze alterations in the amount and behavior of free radicals to understand free radical mechanisms like the thermal conversion of petroleum [67]. Moreover, the significant parameters of ESR like *g*-value and linewidth as described above can be used to predict the ESR spectra of the fractions through the analysis of peak area, position, and shape, which in turn assist in uncovering the concurrent variations in radical concentration, types, and coke yield to clarify the chemical process of coke generation during these reactions. Thus, the wide range of applications of ESR spectroscopy in the field of petroleum demonstrates the utility of this technique in analyzing heavy fractions, such as oil sands bitumen, asphaltenes, vacuum residue, HGO, VGO, coals, and biomass not only for characterization purposes, but also to investigate the free radical mechanisms in thermal reactions as described below.

## 4. In-Situ Free Radical Analysis during Thermal Cracking Using ESR

As it is described earlier, there is a strong correlation between free radicals and coke production. This is highlighted in studies conducted on stable radicals under pyrolysis in unconventional oils including crude oil, coal, and biomass. In this section of the manuscript, we exhaust the literature that used ESR to study the in-situ free radical concentration during the pyrolysis of unconventional crude oils, coal, and biomass for purposes related to mechanistic understanding. Table 1 below represents the list of research conducted on heavy oils in the past decade.

**Figure 9:**
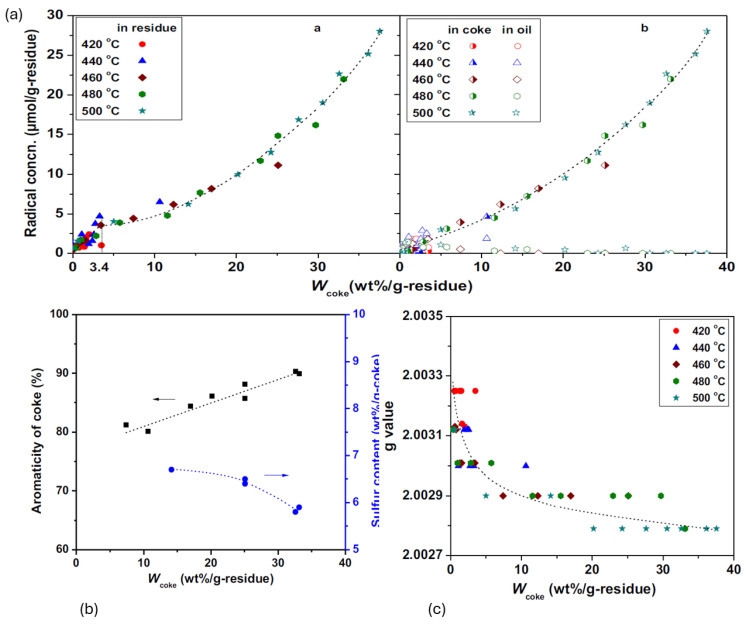
The graph shows that free radical reactions during thermal cracking cause the formation of high aromatic compounds (**a**) shows the variation in stable radical concentration in oil, residue and coke, (**b**) represents the direct proportion of aromatics in coke, (**c**) represents the *g*-value at different coke weight %/g of residue. This figure was regenerated based on a figure from Shi et al. 2019 [6].

We have previously postulated that free radical addition reactions taking place during thermal cracking, are the major reason for asphaltenes and coke formation [32]. The study by Shi et al. [6] confirms this hypothesis as it shows that coke condensation and oil cracking are involved in the generation of stable radicals. When the coke level is less than 3.4 wt%, half of the stable radicals are present in the coke oil, whereas when the coke content is more than 8.0%, nearly all stable radicals are present in the coke as in (Figure 9a). In addition, the radical concentration increased as the weight of coke increased. This behavior of free radicals was also observed during the pyrolysis of coal tar [83]. In addition, the coke’s condensation is accompanied by the loss of heteroatoms like sulphur atoms and the increase in coke aromaticity at high coke percentages (i.e., 8 to 33 wt%). Furthermore, the stable radicals’ *g*-value and linewidth in coke are correlated with the coke wt% and show a linear and exponential correlation, respectively. This reflects the structural variation of coke. In fact, line width reduces from approximately 0.6 during initial coke generation to below 0.4 when coke wt% is 37%. It reaches 0.55 when the aromaticity of coke reaches 80%. However, when the aromaticity of coke reaches 90% (Figure 9b), the ΔH_pp_ value changes to 0.43. Reduction in linewidth as the percentage of coke weight increased shows the increase in distance between free electrons and the reduction of heteroatoms and sulphur content which indicate high aromaticity and structure change of coke. A rapid decline in the *g*-value from approximately 2.003 to 2.0029 for W_coke_ below 10%, and a gradual decline from 2.0029 to 2.0028 for W_coke_ between 10% and 37% (Figure 9c). The reduction in the *g*-value can be attributed to a variety of factors such as the loss of heteroatoms, whereas condensation of aromatics has the potential to increase *g*-value.

**Figure 10:**
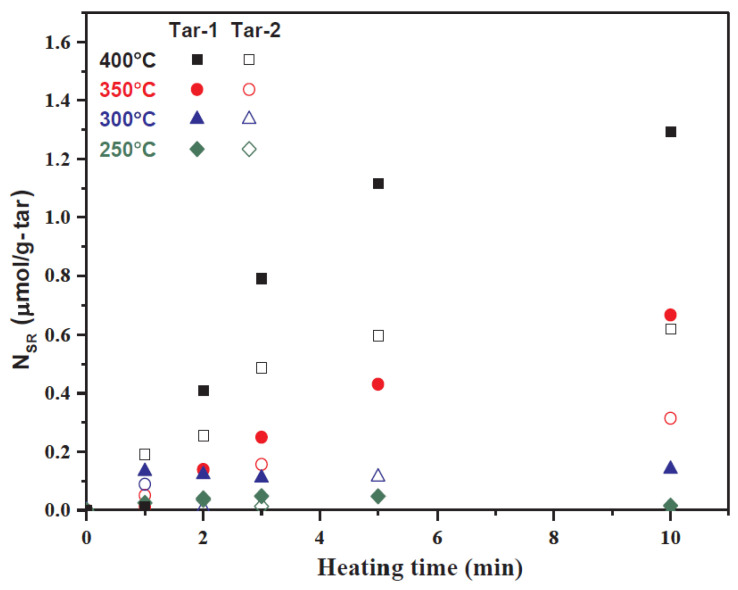
Stable radical concentration (N_SR_) is higher in Tar-1 as compared to Tar-2 when subjected to thermal cracking from 250 to 400 °C. This figure is extracted from the reference [20].

The study by Wang et al. [80] and Zhang et al. [20] confirms the previously discussed inter-connection of free radicals, asphaltenes, and coke production as it was found that a higher amount of asphaltenes and poly-aromatic structures have higher amounts of free radical concentrations. This was also correlated with higher temperatures that initiate bond cleavage [20]. In addition, coke undergoes condensation reactions of large radical fragments produced during the pyrolysis, resulting in agglomeration and increases in the radical concentration. In fact, the variations in the radical quantity indicate the occurrence of condensation processes within the asphaltenes molecules and between the radical fragments [80]. A higher asphaltenes content in tar was also shown to cause more bond cleavage that increases radical concentration during cracking of different tars. These observations align well with previous studies done on coal [83,84]. The magnitude of radical concentration of heavy tars feedstocks suggest that their coking is much lower in free radical concentration than the tars produced by coal and biomass. When comparing stable and reactive radicals, few free radicals survived during coupling and converted into stable ones. Due to this reason, stable free radicals are three orders of magnitude less than the reactive radicals and correlate linearly with the highly reactive radicals in quantity. Thus, the results depicted that higher radical concentration is a comprehensive parameter causing the increase in the cracking and coking behavior of heavy tars, which indicates that Tar-1 is more likely to coke than Tar-2 as shown in (Figure 10) [20].

Interestingly, when studying the SARA fractions of the vacuum residues, the Asp fraction exhibits a higher degree of interaction within free radicals and a high condensation rate as compared to other fractions during cracking. The Sa (Saturates) and Ar (Aromatics) fractions exhibit lower reactivity and produce fewer radicals compared to the Re (Resins) and Asp (Asphaltenes) fractions. Other SARA fractions do not produce adequate quantities of radicals during cracking; therefore, dilution of Asp with other fractions has no significant effect on the formation of radicals because of the lack of interaction among free radicals to condense. This reflects the dilution effect that was previously studied [57] showing that the solvent may play a role in moving the dynamic equilibrium of free radicals leading to either their dissociation into pairs or their association into a neutral molecule.

A study was conducted on soft and hard coke obtained from the pyrolysis of heavy oil [81]. Results showed that the hard coke’s radical concentration moderately increases at 400 and 420 °C reflecting cracking and condensation of free radicals. At a coke content of 60 wt%, the radical concentration reaches 45 µmol/g in hard coke. On the other hand, in soft coke, both the mass and radical concentration increase with time in temperature ranges of 350–420 °C. However, radical concentration declines at approximately 17 µmol/g in soft coke when total coke content is around 13 wt%. The observed behaviors suggest that soft coke formation and structural condensation via cracking are occurring simultaneously. Over time, soft coke exhibits an opposite trend between mass and free radical concentrations. In other words, a decrease in mass was noticed by an increase in stable radical concentration that represents the transformation of soft into hard coke via cracking and condensation in the final stage. Moreover, the line width of both cokes decreases with time and under high temperature. Line width of hard coke appeared narrower than soft coke indicating high stable radical concentration [81].

To better understand the impact of various free radical concentration on the mechanistic changes during pyrolysis Hernandez et al. [82] defined different temperature points that signify a major mechanistic change: (i) the cusp temperature, that is, the point at which free radicals start to increase in concentration by bond cleavage of the heavy structures and radical chain fragmentation within the samples (ii) the valley temperature at which free radical concentration stops increasing, and (iii) the second cusp temperature is at which free radical concentration starts decreasing because of coupling reactions. In their study, a transition is noted at 450 K. Free radical coupling was observed at a low temperature in asphaltenes fractions than the actual oil sample and the formation of free radicals was observed at high temperature range due to reduced radical recombination. It was also observed that there were stronger free radical interactions in the unfractionated samples compared to the fractions and trapped compounds as they required a lower temperature to initiate free radicals. This is due to the fact that unfractionated samples are higher in poly-aromatic structures.

The research conducted on heavy oil fractions as mentioned above summarizes the free radical behavior and associated mechanisms such as condensation and polymerization that is associated with the formation of heavy compounds during the thermal cracking of different heavy oil fractions. Though very limited research has been conducted on heavy oils in the last decade, it is now established that (i) condensed polyaromatic rings, reflecting structures found in asphaltenes and coke, have the highest free radical concentrations. (ii) the initiation of free radicals changes with various feedstocks depending on their properties, (iii) free radical concentration fluctuates during pyrolysis reflecting the various propagations steps taking place (i.e., decrease in free radicals with coupling, increase in free radical with bond cleavage and increase in free radicals with coke formation due to the formation of stable sterically hindered free radicals).

The literature on behavior of free radicals on oil shale which is also a source of unconventional oils is presented in (Table 2) below.

**Figure 11:**
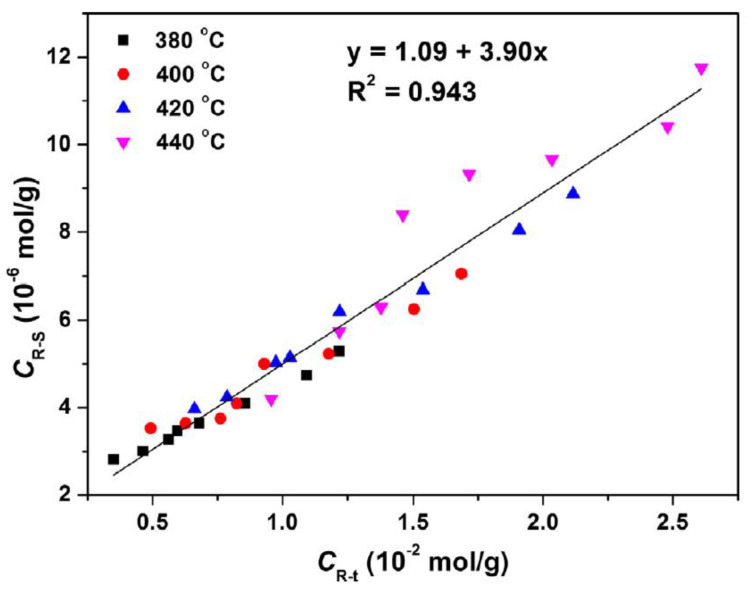
This graph illustrates that increase in total radical concentration (C_R-T_) leads to an increase in stable radical concentration (C_R-S_) with the slope of 3.90 × 10^−4^ and the reciprocal of the slope is shows that 2500 C_R-T_ involved in the formation of one C_R-S_, which condensed further to form coke. Figure extracted from the source [21].

Similar to heavy oils, a study on Huadian oil shales, investigating the amounts of total radicals, including active and stable radicals, shows that the distribution, quality, and composition of pyrolysis products are dependent on the free radical propagation reactions [21]. As discussed in previous literature, the temperature increase boosts bond cleavage and the formation of free radicals, resulting in an increase in total free radical concentration in the product. In fact, active radicals condense to form large organic molecules and stable radicals. According to literature, coupling of about 2500 active radicals are involved in the generation of one stable radical. After the formation of stable radicals, their condensation reaction starts to form heavy products such as char and coke. Results represented a linear relationship between the increase in total radical concentration and stable radicals and the correlation of stable radical formation with coke generation as depicted in (Figure 11).

In addition to confirming the previous observation, the study by Zhao et al. [85] on active and stable radicals in the organic matter (OM) of eight shale oils under pyrolysis shows that at 420 °C, 15.1–24.3 mmol/g of active radicals are produced for 12 min, of which half are produced in 2 min. At 12 min, YLOM (Yilan organic matter) sample reaches a maximum of 2.12 × 10^−5^ mol/g, whereas for MAOM (Morocco Organic matter) 3.23 × 10^−6^ mol/g radical concentration was observed. This indicates that the stable radicals undergo rapid formation in the samples with higher aromaticity. Results depict that the aromaticity of OM in oil shale influences the concentration of free radicals exponentially. In addition, stable radical concentration exhibits an increase with the increase in coupling rate of active radicals within the 10^−6^–10^−5^ mol/g as the pyrolysis time progresses. Coupling reduces the quantity of active radicals produced by bond cleavage to below 0.1%, which causes the formation of stable radicals [85]. Further, the condensation reactions between stable radicals generate the formation of heavy compounds like coke that contain about 99.9% of stable radicals during the thermal reaction of oil, or coal as also described in [21]. The study by Wang et al. confirms the previous observations on oil shale and heavy oils [86]. However, it also shows a very crucial application for the measure of free radicals in oil shale and its pyrolyzed products, namely asphaltenes and semicoke, that is asphaltenes being an intermediate product for coke formation. The free radical concentration of asphaltenes exhibits a positive correlation with temperature till 430 °C, and declines as the temperature rises above 430 °C. This suggests that thermal bitumen functions as an intermediate product in the pyrolysis of oil shale. There was also a significant variation in the *g*-value of semicoke across the entire temperature range indicating that the pyrolysis process causes a significant structural and environmental transformation in semicoke. As the temperature rises, the linewidths of shale oil and its pyrolyzed products (i.e., semicoke and asphaltenes) vary as a result of the interactions between the hydrocarbon compounds and the free radicals via spin−spin and spin−lattice [86].

Studies summarized above show limited data regarding the research on free radicals and its associated mechanisms in oil shale. It shows only the relationship between stable and active radicals as well as the link between aromatic compounds and free radical formation and concentration.

The research conducted on biomass for the study of free radicals in the past decade is represented in (Table 3).

To further investigate the role of free radicals, studies were also conducted on various biomass materials. A study on free-radical behavior during the thermal decomposition of lignin, cellulose, and hemicellulose at 350, 400, and 440 °C, also analyzes the results after the addition of 9,10-dihydrophenanthrene (DHP) as a hydrogen donor [87]. It is well established that hydrogen donors prevent the condensation of free radicals and thus reduce the formation of heavy compounds [80]. The behaviors of cellulose and hemicellulose exhibit a great degree of similarity, but they diverge significantly from the behavior of lignin. Lignin shows high bond cleavage (0.89 × 10^−2^) at first 10 min of pyrolysis as compared to cellulose and hemicellulose which indicates that it consists of a higher amount of weak covalent bonds as compared to cellulose and hemicellulose. When the biomass is pyrolyzed, the radical fragments combine to create heavy products like coke that effectively capture radicals, especially in the case of lignin. These findings agree with another study on walnut shell (WS) and corncob (CC) in which the free radical concentration in the WS tar is twice that of the CC tar due to the greater lignin content in WS [90].

In another context, it was found that in the presence of DHP, the free radical concentration of lignin, cellulose, and hemicellulose increases to 2.1 × 10^−5^, 0.7 × 10^−5^, and 0.7 × 10^−5^ mol/g after 3 min, but also the rate of free radical chain coupling reduced in all of the three biomass samples. In fact, 99.9% of the radicals produced during pyrolysis are combined with hydrogen provided by DHP [87].

**Figure 12:**
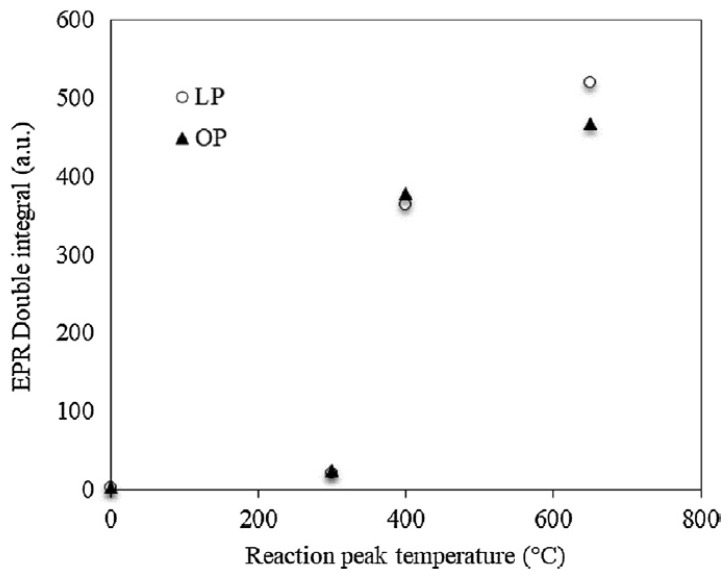
Each point represents radical concentration by the double integral of ESR curves at different temperature in LP and OP samples from the reference [88].

In another study, they examine the free radical concentration in solid byproducts generated during the pyrolysis of citrus waste [88]. Chars were produced through the slow pyrolysis of orange and lemon pulp (OP and LP) in a horizontal batch reactor with a temperature range from 200 to 650 °C (50 °C/min^−1^). ESR is used to access the physio-chemical evolution of radicals in biomass during pyrolysis. Samples at 300 °C showed the same spin population as the raw sample indicating that there is a relatively small number of covalent bonds cleaved at that temperature. The free spin population of biomass increases as the temperature rises above 250 °C. This results in the initiation of bond breaks in a covalent matrix whereas above 400 °C, the rate of increase diminishes. These results support the findings reported in [92], which showed that dissociation of covalent bonds begins at approximately 350 °C and that the samples’ reactivity is greatest between 300 and 500 °C. Thus, it is anticipated that a greater quantity of aromatic fragments may be generated during lignin decomposition at temperatures above 500 °C. The values measured for feedstock and char samples produced at temperatures of 300, 400, and 650 °C are illustrated in (Figure 12). It is significant to note that free radicals concentration is higher in LP because of high lignin content which contain weaker covalent bonds and high aromatic fragments. Results depicted that determining the temperature range during which the population of these species increases dramatically, assist in the study of the evolution of the samples and its overall reactivity which is significantly influenced by free radical concentration. The reactions between vapors produced during the pyrolysis and the solid surface of chars lead to the formation of more chars due to the presence of free radicals bounded in existing char structures. [88] In a similar context, during the pyrolysis of six various biomass, it was found that the radical concentration increased prior to 400 °C, then reached the highest concentration at approximately 600 °C. It then declines rapidly between 600 and 800 °C, ultimately reaching its minimum at 800 °C [91]. Interestingly, the *g*-value decreased from 2.0033 to 2.0024 with the increase in pyrolysis temperature reflecting a change in the radical centre or radical environment. In the case of biomass, the *g*-value reflects both carbon and oxygen-centred free radicals. Char produced from all the biomass samples exhibit broader line width at 350 °C and 700 °C. The varieties of radicals present in biochars was influenced by the type of the biomass feedstock and the quantity and type of substitutional groups attached to aromatic rings present in it [91].

The coking of rice husks was studied and the findings suggest that the process of bio-oil coking in pyrolysis can be categorized into three distinct phases, each exhibiting unique properties [89]. During the initial phase of coking, a considerable quantity of stable free radicals is produced and bound retained within coke structures. Following this, during the final phase, the precursors of coke are exhausted, the nascent coke condenses gradually via thermal reactions, and the concentration of stable free radicals in the nascent coke increases gradually. The correlation between coke formation and stable free radical generation is evident, suggesting that stable free radicals are primarily generated concurrently with coke following the induction phase in bio-oil pyrolysis.

Limited research is found on the analysis of free radicals during the thermal conversion process of biomass. All cited literature agrees with the findings previously reported in oil shale and heavy oil. Additionally, it was unambiguously stated that the lignin portion of the biomass is responsible for most of their free-radical content due to the nature of its structure that differs from cellulose and hemicellulose specifically in terms of aromaticity and ease of bond cleavage.

Finally, this portion reviews literature from 2014 to 2019 and compared it with the latest research done from 2020 to date on coals after the review paper of Zhou et al. [17] was written. Although the research after 2020 is limited, the results show alignment with findings from existing research done on coals as represented in (Table 4). It also shows that a reasonable amount of research was conducted on coals as compared to heavy oils, oil shales, and biomass. The literature below represents the research conducted on in-situ study of free radical reactions on various aspects in coals such as cracking in the presence of hydrogen donor, under the presence of various gases, different types of coke and tars produced and its link with free radicals during thermal reactions.

As shown in the “outcome” column of Table 4, all studies on coal elucidate similar conclusions to those of heavy oil and oil shale specifically in terms of dependence of free radical concentration on heavy products formation and bond cleavage as well as the decrease of *g*-value with increasing temperature.

In addition to these observations, various researchers analyzed the behavior of free radicals in coke at various residence times, temperature and in the presence of different gases for a better understating of free radical mechanisms. A study on the role of the free radicals during the pyrolysis of coal/char under the influence of different temperatures and two different gases showed that between 400 °C and 500 °C, the levels of free radicals significantly rise, resulting in the release of volatile substances [96]. The levels of free radicals consistently rise below 600 °C for chars that have undergone pyrolysis in both nitrogen (N_2_) and carbon dioxide (CO_2_) environments. As the temperature increases, the concentrations of spin decrease significantly in the chars at 800 °C. There are larger concentrations of free radicals formed in the N_2_ atmosphere compared to the CO_2_ atmosphere. It was shown that oxygen-containing radicals and aromatic clusters are the significant radical groups that play a key role in the thermal destruction of coal particles.

In a recent study, not covered in the previous review paper, the main radical source in tars was attributed to the coke fractions. This was observed during the coking behavior of volatiles in three low rank coals named Naomaohu (NMH), Zhundong (ZD), and Zichang (ZC) [19]. Proportional correlations exist between the radical concentration and molecular weight of tar and coke yield. The radical concentration in ZC volatiles is significantly greater than that in ZD and NMH volatiles, showing that the radical concentration in volatiles is positively correlated with the high presence of aromatic compounds and yield of coke, which increases tar production.

Another recent study analyzed coal co-pyrolysis, showing the interactions occurring within free radicals formed during the co-pyrolysis process of coal and biomass also known synergistic effect [99]. The synergic effect between these two feedstocks can be either positive or negative depending on the cracking temperature, feed type, and mixing ratios. Positive synergistic effect was analyzed in the following study as the concentration of free radicals in the samples increased as the temperature increased [99]. Prior to pyrolysis, the free radical concentrations of Hulunbeier lignite (HLBE) coal, walnut shell (WS), and pine for pyrolysis were as follows: 6.2 × 10^18^, 1.1 × 10^17^, and 1.7 × 10^17^ spins/g. Results show that the radical concentration of co-pyrolysis tar of coal/WS was more than that in individual samples, because of the additional reactions by volatiles. The co-pyrolysis tar is higher in radical concentration and unstable because of volatiles additional reactions which seemed to be absent in individual tars which indicates the positive synergy between coal and biomass. The increase in coke production is due to the combination of volatile and stable radicals and the failure of volatile radicals to escape from the heavy substance structures. A negative synergistic effect of the free radical behaviors was observed during the co-pyrolysis of a biomass mixture consisting of pine wood sawdust and low-rank lignite coal [58]. The study shows that coal char is more unstable than the chars produced by co-pyrolysis of biomass and coal. Co-pyrolysis char consists of low concentration of free radicals which shows the negative synergistic effect of the addition of biomass with coal.

The research on thermal reactions involving coal as feedstock seems to be more established than other unconventional feedstocks, especially in heavy oil fractions. Still, there is a gap in understanding the behavior of free radical formation during the co-pyrolysis of coals with biomass. By understanding the mechanisms involved in the positive and negative synergistic effects during co-pyrolysis upon the use of different coal and biomass samples, researchers will be able to enhance the coal conversion process without affecting the product quality.

## 5. Conclusions and Future Perspective

In this article, we highlight the importance of in-situ ESR measurements for mechanistic studies of non-catalytic thermal cracking of various unconventional resources including heavy oils, oil shales, biomass, and coal. We first provide an overview of the free radical reactions that can take place and their impact on the various types of products obtained. The drawback of coke formation by addition reactions is well established necessitating a full understanding of the mechanisms of these reactions to limit the formation of such undesirable products. We also briefly introduce ESR spectroscopy and indicate how the *g*-value and line width could be used to understand the free radical behavior in various chemical reactions involving a free radical mechanism.

In this work, we critically review the literature in the last decade, conducted on thermal cracking of heavy oil, oil shales, coal, and biomass using ESR spectroscopy.

The motivation behind understanding such free radical mechanisms are as follows: (i) the necessity of use of unconventional resources due to the depletion of conventional ones, (ii) the need for upgrading unconventional sources in order to refine them later and convert them into valuable products, and (iii) the formation of undesirable heavy compounds by free radical addition reactions that lead to product instability and equipment fouling. In other words, efficient conversion processes are required for the cracking of unconventional oils to possibly reduce the formation of heavy compounds. It is necessary to get insights into the various pyrolytic conditions and mechanisms that affect the initiation, propagation, and termination steps.

It was found that very limited research has been established on the in-situ study of free radicals’ behavior and its relationship with coke formation through ESR in unconventional sources of hydrocarbons. Most of the literature covered in the summarized tables agree on the following conclusions: (i) condensed polyaromatic rings, reflecting structures found in asphaltenes and coke, have the highest free radical concentrations, (ii) the initiation of free radicals changes with various feedstocks depending on their properties, (iii) free radical concentration fluctuates during pyrolysis reflecting the various propagations steps taking place (i.e., decrease in free radicals with coupling, increase in free radical with bond cleavage and increase in free radicals with coke formation due to the formation of stable sterically hindered free radicals), (iv) the *g*-value and linewidth are changing with the free radical formation due to bond cleavage or to free radical combination during condensation to coke, reflecting changes in the nature of free radicals, (v) the lignin portion of the biomass is responsible for most of their free radicals content due to the nature of its structure that differs from cellulose and hemicellulose specifically in terms of aromaticity and ease of bond cleavage, and (vi) lignin pyrolysis leads to the highest coke formation compared to its counterpart (i.e., cellulose and hemicellulose).

A number of research gaps were identified in terms of the use of ESR for in-situ mechanistic study of various unconventional sources.

In the case of heavy oils and oil shales, the following should be further investigated: (i) the interactions between the stable radicals with total radical concentration in the oil during pyrolysis, (ii) behavior of free radicals in heavy oil pyrolysis under the presence of various gases to track the rate of additional oxidation reactions associated with thermal reactions, (iii) variations in free radical concentration in different types of coke (e.g., soft and hard) produced during heavy oil pyrolysis, (iv) deeper analysis of variations in *g*-value which can give information about the changes in coke structure, (v) tracking free radicals when co-feeding different hydrogen donors in different heavy fractions to gain insights on how this impacts the rate of condensation reactions, (vi) tracking the free radical changes with the asphaltene contents in different heavy oil feeds at different temperatures.

In the case of biomass, as free-radical mechanisms and reaction patterns are highly dependent on biomass feed and pyrolysis conditions, it is necessary to: (i) analyse the rate of bond cleavage and formation of stable radicals in individual biomass feed and co-pyrolysis products, (ii) investigate the role of high lignin content in increasing free radical’s production and their reactivity, and (iii) the analysis of free radical behavior under various pyrolytic conditions.

In the case of coals, the gap was mainly observed in explaining the relationship of positive and negative synergistic effects during the co-pyrolysis upon using different coal and biomass feed. This reason compromised the rate of thermal conversion process during coal co-pyrolysis. Moreover, a detailed analysis of *g*-value and line width in relation to coke structure in different feeds should be addressed. Simplifying the ESR spectra through the removal of instrumental noise in spectral lines using data deconvolution techniques will provide a clear understanding of radical behavior and key parameters during data interpretation.

## Figures and Tables

**Table 1:** List of studies that employed ESR for in-situ study of free radicals behavior and associated mechanisms of pyrolysis reactions of various heavy oils.

Entry	Domain (Thermal Reactions)	Feed	Temp.	Time	Outcome	References
1.	Behavior of coking and stable radicals formation during thermal reaction of an atmospheric residue	Atmospheric residue	460 °C	20, 40, and 60 min	Stable radicals exist in oil when coke content was lower than 3.4%. 90% of the total stable radicals was found in the coke when coke contents exceeded 8.0%. Linewidth shows steady reduction while *g*-value reduces exponentially with the increase in W_coke_	[6]
2.	Radicals and coking behaviors during thermal cracking of two vacuum resids and their SARA fractions	Anqing (AQ) & Qingtao (QD) Vacuum residues and their SARA fractions	400–460 °C	1.5, 3.0, 5.0, 7.5, 10.0, 15.0, 20.0, 30.0, 40.0, and 60.0 min	Asphaltenes agglomeration increases radical concentration and coke production. The (QD-VR) has a high Asp fraction and poly-aromatic structures, thus higher in radical generation.	[80]
3.	Behaviors of coking and stable radicals of heavy oil during thermal reaction in sealed capillaries	Heavy Oil (soft and hard coke)	250–500 °C	0.5–40.0 min.	Hard coke formed at 400–420 °C whereas soft coke was observed at 350–420 °C. Radical concentration was increased to 45 µmol/g and 17 µmol/g in both coke, as temperature rises. Line width of hard coke was narrower than soft coke indicating high radicals concentration.	[81]
4.	Bond cleavage and reactive radical intermediates in heavy tar thermal cracking	heavy tar samples	250–400 °C	1.0–10.0 min	Co-relation between reactive and stable radicals was studied. TAR-1 and 2 start coking at 300 °C due to cracking and radical fragmentation. Stable radicals are higher in tar-1 than in tar-2 because of high condensation rate in tar-2.	[20]
5.	Electron paramagnetic resonance study of the fractions and trapped compounds in asphaltenes of Merey heavy crude oils and its vacuum residue	Merey heavy crude oil and its (VR)	90–600 K	-	Free radical concentration started to increase at 450K in all samples. Fractionated samples A1 and A2 (ASCM) contain higher free radical concentrations. Whereas, A1 and A2 fractions of vacuum residues (ARVM) exhibit strong free radical interactions which lower the concentration.	[82]

**Table 2:** List of studies that employed ESR for in-situ study of free radicals behavior and associated mechanisms of pyrolysis reactions of shale oils.

Entry	Domain (Thermal Reactions)	Feed	Temp.	Time	Outcome	References
1.	The bond cleavage and radical coupling during pyrolysis of Huadian oil shale	Huadian acid-treated oil shale	380–440 °C	2 min	The number of stable radicals shows a linear relation with that of total radicals generated during pyrolysis. one stable radical is formed by 2500 active radicals generated from HDOM	[21]
2.	A study on average molecular structure of eight oil shale organic matters and radical information during pyrolysis	Organic matter (OM) of 8 oil shale samples from different origin	420 °C	12 min	Samples with higher aromaticity have higher concentrations of stable radicals. The aromaticity determines stable radicals, which ranges from 4.22 × 10^−7^ to 8.10 × 10^−6^ mol/g. Active radicals are volatile and couple to form stable radicals.	[85]
3.	Effect of Temperature on the ESR Properties of Oil Shale Pyrolysates	Oil shale and pyrosylates (semicoke& thermal bitumen)	370–550 °C	20 min	Free radical concentration is higher in semicoke and thermal bitumen than in shale oil. Furthermore, it is observed that the *g*-value of semicoke lowers significantly as the temperature rises. Due to the increasing proportion of aromatics, the *g*-value of bitumen decreases to 2.00301. From largest to smallest, the ESR linewidths consist of semicoke, thermal bitumen, and shale oil, in that order.	[86]

**Table 3:** List of studies that employed ESR for in-situ study of free radicals behavior and associated mechanisms of pyrolysis reactions of Biomass.

Entry	Domain (Thermal Reactions)	Feed	Temp.	Time	Outcome	References
1.	Cleavage of covalent bonds in the pyrolysis of lignin, cellulose and hemicellulose	lignin (kraft lignin) and cellulose	350–440 °C	10 min	Radical concentration was higher in lignin as compared to other samples in the presence and absence of DHP. Moreover, the presence of DHP inhibits free radical condensation thus reduces coke weight which suggests the link between free radicals and coke formation.	[87]
2.	Free radicals formation on thermally decomposed biomass	Lemon and orange pulp	200–650 °C	-	Free radical formation initiates from 330–350 °C. Lemon pulp (LP) shows higher radical concentration than orange because high lignin content in LP causes the formation of aromatic structures.	[88]
3.	Identifying the coking of bio-oil in pyrolysis: An in-situ ESR investigation	Rice husks	250–350 °C	2–10 min	After the induction period of 8 min coke starts formed and the radical concentration also increases. In the final phase, radical concentration increased by coke condensation, not by the coke formation. It shows the strong relation between free radicals and coke.	[89]
4.	Understanding the stability of pyrolysis tars from two biomass in a viewpoint of free radicals	walnut shell (WS) & corncob (CC)	293–873 K	4 h–2 weeks	WS tars and its aqueous liquid sample contain a higher amount of stable radicals than in corncob (CC) tar and its liquid sample. Free radical concentration was observed to be highly associated with weak covalent bonds and coke formation. It causes poor stability and quality.	[90]
5.	Formation and evolution mechanism of persistent free radicals in biochar during biomass pyrolysis: Insights from biochar’s element composition and chemical structure	rice straw, rice husk pine wood, bamboo powder, corncob, and coconut shell	350–800 °C	0–60 min	Free radical concentration increases between 400–600 °C and then reduces at 800 °C. The *g*-value and linewidth decline as the temperature rises It shows that the Biochar produced during pyrolysis contain carbon and oxygen-centered radicals.	[91]

**Table 4:** List of studies that employed ESR for in-situ study of free radicals behavior and associated mechanisms of pyrolysis reactions of coals.

Entry	Domain (Thermal Reactions)	Feed	Temp.	Time	Outcome	References
1.	The radical and bond cleavage behaviors of 14 coals during pyrolysis with 9,10-dihydrophenanthrene	14 coal samples	440 °C	0.25 min	The quantities of active radicals are 3 orders of magnitude higher than stable radicals. Stable radicals were higher in coal with aromaticity. While, bond cleavage was difficult with the increase in coal rank.	[93]
2.	Formation of radicals in coal pyrolysis examined by electron spin resonance	Mataihao bituminous coal (MTH)	24–800 °C	-	Free radical concentration was divided into 3 phases in coal. As temperature increases from 250 to 350 °C, the radical concentration elevates as the *g*-value decreased. In phase 3, formation of aromatic radicals occurred above 380 °C.	[94]
3.	Coke formation during thermal reaction of tar from pyrolysis of a subbituminous coal	subbituminous coal	600 °C	2.1, 6.3, 12.6 and 21.0 s (RT)	Free radical concentration in tars increased by increase in temperature and the residence time at each temperature. It contributes in coke formation. Thus, coke content can be measured by radical concentration in tars and shows a correlation.	[84]
4.	Behaviors of coking and kinetics of volatiles’ reaction during coal pyrolysis in a two-stage reactor	Low rank coals (Naomaohu, Zhundong, and Zichang)	440–700 °C	6.9, 4.2, 3.0, 2.2, or 1.5 s	Radical concentration increases with temperature and residence time. ZC coke contains higher concentration of free radicals than other samples because of high aromatic structures. Strong relationship between free radicals and coke was observed.	[19]
5.	Analysis of tars produced in pyrolysis of 4 coals under various conditions in a viewpoint of radicals	Hulunber coal (HLBE), Bulianta coal (BLT), Buertai coal (BET), and Daliuta coal (DLT)	350–540 °C.	2.4–1.1 s (RT)	As temperature increased, tars produced by DLT sample are higher in radical concentration. In contrast to other samples because of high carbon %. Thus, DLT tar is more reactive. Moreover, increase in residence time also enhance bond breakage and radical concentration.	[95]
6.	Influences of particle size, ultraviolet irradiation and pyrolysis temperature on stable free radicals in coal	Shenhua and Nei Monggol coals	300–800 °C	-	Bond breakage start at 350 °C, and at 400–500 °C there was significant rise in radical concentration in samples. It start contributing in coke and char formation. Moreover, radical concentration and change in their types in samples pyrolyzed in N_2_ was greater than the sample pyrolyzed in CO_2_ environment.	[96]
7.	Behaviors of coking and radicals during reaction of volatiles generated from fixed-bed pyrolysis of a lignite and a subbituminous coal	Shenmu coal (SM) and Hulunbeier (HLBE)	400–700 °C	1.5–6.9 s (RT)	Increase stable radical concentration, and decrease *g*-value and line width as temperature increased was observed in both samples. It showed the structural change in coke due to condensation of aromatic rings.	[97]
8.	Behaviors of radical fragments in tar generated from pyrolysis of 4 coals	(HLBE), (BLT), (BET) (DLT) coals	293–773 K	0.5–4 h	Radical formation was observed in tars of 4 coals. It increased from 10^17^ spins/g to 10^19^ spins/g under temperature range from 623 K to 723 K. it induces coke formation which further increase radical concentration in tars and contribute to its high reactivity.	[83]
9.	Characterization of coke formed during thermal reaction of tar	subbituminous coal	300–500 °C	40 min	Free radical concentration increased in coke S and coke D as temperature rose. Coke D exhibit low *g*-value and line width as compared to coke S due to higher condensation of aromatic compounds.	[98]
10.	Interactions between free radicals during co-pyrolysis of lignite and biomass	(HLBE coal) & biomass (WS and pine)	~380–600 °C	5 min	Free radical concentration was increased at 550 °C in all samples from their initial values. Highest concentration was observed in coal/WS co-pyrolysis than in coal/pine at same pyrolysis conditions.	[99]
11.	Effect of Microwave and Thermal Co-pyrolysis of Low-Rank Coal and Pine Wood on Product Distributions and Char Structure	Mississippi coal, lignite coal, and Yellow pine sawdust, wood pellets	550 °C	2 h	Free radical concentration elevates as temperature rises in the chars of coals and co-pyrolytic samples. Carbon-centered radicals were in large amount than oxygen-centered. Moreover, *g*-value of coal char is greater than *g*-value of coal/biomass char. It shows the instability of coal char.	[58]

## Data Availability

The datasets used and/or analyzed during the current study are available from the corresponding author on reasonable request.

## Nomenclature

C-O	Carbon-Oxygen.
C-S	Carbon-Sulfur.
C_al_-C_al_	Carbon_Aliphatic_-Carbon_Aliphatic._
C_ar_-C_al_	Carbon_Aromatic_-Carbon_Aliphatic._
DHP	9:10-dihydrophenanthrene.
FTIR	Fourier-Transform Infrared Spectrometry.
NMR	Nuclear Magnetic Resonance.
IR	Infrared Spectroscopy.
ESR	Electron Spin Resonance.
H/C	Hydrogen to Carbon ratio.
α–C_al_	Carbon_alpha_-Carbon_Aliphatic._
β–C_al_	Carbon_beta_-Carbon_Aliphatic._
C_ar_–C_al_–C_al_	Carbon_Aromatic_-Carbon_Aliphatic_-Carbon_Aliphatic_ Chain.
C_al_–O	Carbon_Aliphatic_-Oxygen.
C-C	Carbon–Carbon.
±1/2	Spin up and spin down transition state of free electron.
HGO	Heavy Gas Oil.
VGO	Vacuum Gas Oil.
VR	Vacuum Residue.
AR	Atmospheric Residue.
AQ	Anqing vacuum residue.
QD	Qingtao vacuum residue.
SARA	Saturates: Aromatics, Resins, Asphaltenes.
Sa	Saturates.
Ar	Aromatics.
Re	Resins.
Asp	Asphaltenes.
ΔH_pp_	Linewidth.
Wt%	Percentage weight.
µmol/g	Micro mole per gram.
°C	Celsius.
OM	Organic Matter.
W_coke_	Percentage weight of coke per gram of residue
ASCM	Asphaltenes from the Merey crude oil
ARVM	Vacuum residue of asphaltenes from the Merey crude oil
HDOM	Huadian Organic Matter.
C_R-T_	Total radical concentration.
C_R-S_	Stable radical concentration.
HLBE	Hulunber coal.
BLT	Bulianta coal.
BET	Buertai coal.
DLT	Daliuta coal.
Rt	Residence time.
Mol/g	Mole per gram.
NMH	Naomaohu coal.
ZD	Zhundong coal.
ZC	Zichang coal.
